# “Nothing's changed, baby”: How the mental health narratives of people with multiple and complex needs disrupt the recovery framework

**DOI:** 10.1016/j.ssmmh.2023.100221

**Published:** 2023-12

**Authors:** Joy Llewellyn-Beardsley, Stefan Rennick-Egglestone, Felicity Callard, Kristian Pollock, Mike Slade, Alison Edgley

**Affiliations:** a School of Health Sciences, Institute of Mental Health, University of Nottingham, Triumph Road, Nottingham, NG7 2TU, UK; b School of Geographical and Earth Sciences, University of Glasgow, 8NN, University Avenue, Glasgow, G12 8QQ, UK; c School of Health Sciences, University of Nottingham, Queen's Medical Centre, Nottingham, NG7 2HA, UK; d Nord University, Faculty of Nursing and Health Sciences, Health and Community Participation Division, Postbox 474, 7801, Namsos, Norway

**Keywords:** Mental health, Recovery narratives, Epistemology, Substance use, Homelessness, Sex working, Narrative inquiry, Narrative analysis, Trauma

## Abstract

The dominant narrative in mental health policy and practice has shifted in the 21st century from one of chronic ill health to a ‘recovery’ orientation. Knowledge of recovery is based on narratives of people with lived experience of mental distress. However the narratives of people experiencing structural inequalities are under-represented in recovery research. Meanwhile, uses of recovery narratives have been critiqued by survivor-researchers as a co-option of lived experience to serve neoliberal agendas.

To address these twin concerns, we undertook a performative narrative analysis of two ‘recovery narratives’ of people with multiple and complex needs, analysing their co-construction at immediate/micro and structural/macro levels. We found two contrasting responses to the invitation to tell a recovery story: a *narrative of personal lack* and a *narrative of resistance*. We demonstrate through reflexive worked examples how the genre of recovery narrative, focused on personal transformation, may function to occlude structural causes of mental distress and reinforce personal responsibility in the face of unchanging living conditions. We conclude that unacknowledged epistemological assumptions may contribute to co-constructing individualist accounts of recovery. A critical, reflexive approach, together with transparent researcher positionality, is imperative to avoid the epistemic injustice of a decontextualised form of recovery narrative.

## Highlights

• People with multiple complex needs may tell recovery stories of personal lack.
• People with multiple complex needs may tell stories resisting recovery frameworks.
• Structural determinants of mental health are minimised in the recovery model.
• Unacknowledged researcher assumptions may contribute to an individualist focus.
• Critical reflexivity is vital to avoid decontextualised recovery narratives.

## 1 Introduction

The dominant narrative in mental health policy and practice has shifted in the twenty-first century from one of chronic ill health or incurability to a ‘recovery’ orientation (World Health Organization, 2021). A recovery-based approach is now the most common approach in mental health policy and practice within Global North countries (Barlott et al., 2020), and has also been explored for its relevance to Global South settings (Gamieldien et al., 2020).

Narratives of people with lived experience of mental distress have been central in developing this conceptual basis for mental health policy and practice (Smith-Merry, 2020). People with lived experience are often invited to tell recovery-focused stories in research and practice contexts, as described for example in Jijian Voronka's (2019) critical ethnography of her storytelling activities. However, people experiencing poverty, homelessness, intersecting oppressions (based e.g. on ethnicity, gender, sexuality, disability or class), and other forms of marginalisation remain under-represented in recovery research (Karadzhov, 2021; Morrow and Malcoe, 2017). All of these factors are known social determinants of poor mental health (Alegría et al., 2018). More inclusive research has therefore been called for (Lawrence et al., 2021), so that knowledge of recovery is not based solely on the experiences of those who are relatively well-resourced.

People described as having multiple and complex needs (MCN) are one such under-represented group (Padgett et al., 2016). MCN is a commonly-used term in UK substance misuse services to describe co-occurring issues of homelessness, substance use, crime and mental health problems (Harland et al., 2022). A 2015 study found that in England over 250,000 people a year have contact with at least two of the homelessness, substance misuse and/or criminal justice systems, alongside almost universally present mental ill health and poverty (Bramley et al., 2015). This figure is likely to have risen in a post-COVID landscape (Sher, 2021) and in the context of austerity measures introduced by European and US governments to reduce public spending on welfare, health and social care (Altermark and Plesner, 2022; Harland et al., 2022). The COVID-19 pandemic has focused attention on the extent to which socio-economic and ethnic inequalities influence health (Public Health England, 2020), reinforcing an understanding that physical and mental health inequalities will not be reduced without action on social and structural factors (Stansfield and Shah, 2021).

The narratives of people with MCN provide opportunities to explore the plausible effects of socio-economic contexts on individual lives. Structural effects on mental distress may be harder to discern causally in individual narratives but may be at play in what are often conceptualised as ‘merely subjective’ experiences, such as negative self-concept, hopelessness and passivity (Karadzhov, 2021). The use of lived experience narratives by healthcare researchers, educators and mental health organisations, however, has been problematised by survivor-researchers and other critical scholars (LeBlanc-Omstead & Kinsella, 2022). Politically and ethically questionable practices have been exposed, such as pressure to produce particular kinds of narrative which favour organisational purposes (Kaiser et al., 2020), thus risking forms of epistemic injustice (Fricker, 2007).

Chief among concerns are: (i) that narratives with a focus on the possibility of recovery, central to the history of organising resistance and change in mental health systems, have now become a sought-after commodity by organisations, who may use them to harness funding and support; (ii) that such narratives risk encouraging compliance with, not transformation of services; (iii) that certain types of account tend to be privileged in these contexts, such as ‘inspirational’ and ‘safe’ stories that do not discomfit mental health professionals; and (iv) that such narratives, or their reception in the current political climate, can reinforce an emphasis on individual-level recovery factors such as personal resilience, at the expense of acknowledging structural factors, such as socio-economic status (Costa et al., 2012; Harper and Speed, 2012; Fisher and Lees, 2016; Voronka, 2019; Woods et al., 2019).

In this light, a suggested approach to the sensitive use of lived experience narratives in healthcare is one which remains alert to the nuanced relationship between such narratives and the operation of power in the contexts in which they are shared and heard (Sapouna, 2020). This is also a vital consideration for the use of narratives in mental health *research,* wherein the relationship between research study, researcher and participant may perpetuate paternalistic roles and power divisions central to psychiatric treatment (Russo, 2016; Church, 2013).

Performative narrative analysis offers an approach which is alert to structural contexts. Recovery research based on narratives has largely used thematic (e.g. Brown and Kandirikirira, 2007), structural (e.g. Thornhill et al., 2004) or visual (e.g. Doroud et al., 2022) forms of analysis. These approaches focus on *what* is being told, and *how* narrators are telling their stories. A performative approach explores *why* particular stories may be told (Riessman, 2008; Frank, 2010). It examines storytelling in its immediate, socio-cultural and historical contexts, which provide both opportunities and limitations for the teller (Bengtsson and Andersen, 2020) and the ‘hearer’.

In this article we undertake a close examination of the recovery narratives of two individuals experiencing multiple structural inequalities, elicited within a wider recovery-based research study. We deploy a performative analytical approach, paying attention to emergent epistemes at work as the narrative is co-constructed at immediate/micro and socio-structural/macro levels. We use the study's working definition of recovery narratives: "first-person accounts of recovery from mental health problems, referring to events or actions over a period of time, including elements of adversity/struggle and of self-defined strengths/successes/survival" (Llewellyn-Beardsley et al., 2019). We seek to explore: the kinds of recovery stories people with MCN may tell; how micro and macro-level contextual factors may be shaping their accounts; and what ethical and other issues may arise when eliciting recovery stories for research purposes from people facing multiple socio-structural inequalities.

## 2 Method

### 2.1 Study design

Research was undertaken as part of the Narrative Experiences Online (NEON) study (researchintorecovery.com/neon). 77 participants from four under-researched groups, including people with MCN, were recruited into the wider study, as described elsewhere (Llewellyn-Beardsley et al., 2020). This article is based on two of 10 interviews undertaken with people with multiple and complex needs. All participants could opt to donate their stories elicited during the interview to the NEON online intervention.

### 2.2 Participants and recruitment

Participants with MCN were recruited by JLB through a National Health Service (NHS) substance misuse service and a sex workers' rights organisation. JLB discussed the study with staff, who introduced the study to service users whose stories they felt met the ‘recovery narratives’ definition. Those interested were given further information (Appendix A: Supplementary data), and consenting participants were introduced to JLB by staff on the day.

### 2.3 Data collection

Interviews comprised two parts: an open-ended question designed to elicit a narrative (Riessman, 2008) and a semi-structured topic guide inviting participants to reflect on experiences of telling their stories (Appendix A: Supplementary data). Participants received £20 as an honorarium. Interviews were recorded, transcribed and anonymised. Field notes were written post-interview, including reflexive thoughts on the interviewer's role within the narrative process.

### 2.4 Ethics

Ethical Committee approval was obtained in advance (Nottingham 2 REC 17/EM/0401). All participants provided written informed consent. For participant and researcher wellbeing, we recruited people with MCN via services where staff known to participants were available for pre- and post-interview support if wanted. Mutual assessment of appropriateness to proceed was carried out between participant and staff member immediately prior to interview. Participants were assured they could withdraw at any time. JLB had access to post-interview peer and management support.

### 2.5 Data analysis

Co-analysts JLB and AE conducted a performative narrative analysis (Bengtsson and Andersen, 2020; Riessman, 2008). Narrators are seen as co-constructing their stories in dialogue with (i) micro-level contexts (e.g. research participants managing their stories around perceptions of the interviewer) and (ii) macro-level contexts, wherein the influence of a repertoire of hegemonic narratives existing about them (and ‘people like them’) can be found within personal accounts. We refer to these as *meta-narratives*, defined as socially-sanctioned ways of interpreting experiences which a critical mass of people accept as ‘common sense’ (Hagström and Gustafsson, 2019), and which may shape self-definition and conduct (Sakalys, 2000).

JLB immersed herself in the data by re-reading the 10 interview transcripts and field notes from people with MCN. Candidate narratives for selection from the 10 were discussed with AE. Two were chosen for this in-depth analysis, for two reasons. First, they were illustrative of the kinds of structural determinants of mental distress faced by other narrators with MCN. Second, they provided particularly illuminating examples of issues which may arise from the elicitation of recovery-focused narratives from this group, and two contrasting ways of responding. Other ways in which participants from the wider study group responded to the invitation to tell their recovery stories have been outlined elsewhere (Llewellyn-Beardsley et al., 2020).

JLB and AE devised an analysis process using questions drawn from Bamberg's (2020) integrative and Bengtsson and Andersen's (2020) performative approaches (Appendix A: Supplementary data). After separately analysing each narrative, JLB and AE discussed their findings, noting and reflecting on similarities and differences in approach. A consensus was reached on possible interpretations, and these were written up.

### 2.6 Reflexivity

Since in narrative analysis “the investigator becomes an active presence in the text” (Riessman, 2008: 105), JLB and AE reflected on the experiences and expectations around stories they brought to the analytical process. JLB brought her experience as a queer woman growing up in a working-class community in the 1980s. She was able to disengage from internalised meta-narratives of shame through engaging with counter-narratives of resistance and pride within LGBTQ+ communities (Plummer, 2002), thus bringing to the analysis positive experiences of the power of stories to transform stigmatised identities. Her perspective was informed by her background in literature, sociology and professional youth work, including supporting people with MCN in substance misuse services. She was also informed by lived experience of depression in adulthood, during which she sought out recovery narratives (e.g. articles, memoirs and friends’ experiences) for support, inspiration, and insight into experiences of moving on from despair.

AE brought her experiences as a white middle-class child of a mother diagnosed with bipolar disorder during the 1970s, and a grandmother diagnosed with puerperal psychosis in the mid-fifties. Anxious to avoid the same fate and following the births of her own children, she looked for techniques so she might re-narrate her feared fate. Not content with her own re-narration of motherhood she listened to and published from (paid) working mothers’ stories (Edgley, 2021). This process of re-narration taught her to pay attention to the stories we tell ourselves and to challenge discourses that naturalise (essentialise) and pathologise the effects and affects of political and socio-economic disadvantage.

## 3 Findings

### 3.1 Paul: “things just seem to happen. And I'm not sure why”

Paul is a white British man aged 45–54, a long-time drug user recruited through a substance misuse service, where the interview took place. At the time he was homeless and living temporarily with a friend, having just emerged from what he described as his latest ‘bender’ (UK slang for prolonged period of heavy drug/alcohol use). The first extract (Table 1) is taken from the beginning of the interview.

**Table 1:** Paul's story (Extract 1).

I: Could you tell me about your experiences … of mental health, or emotional issues, and of your recovery, or of … ways you coped, or however you want to phrase it basically? Can you tell me as if it was a story, so as if it had a beginning, a middle, and then kind of like where you are at, or stuff about the future? Thanks! P: Mine is … I get fed up sometimes. I don't know if you call it depression or not, I don't know, and all the time I've always gone onto drugs for that, and it just makes it worse, but it just seems a lot better at the time. And I – mine goes in spells for quite a while, and then just come round, get myself clean again, get back to work and then … I've done it for the last 20 years, exactly the same thing. I just get fed up with the job I'm doing or … anything, anything really. But the only problem is now I'm getting a bit too old for it. So I've just been talking to the staff in here, sorting [a] drug substitute prescription, trying to get myself sorted out that way really. I mean it started when erm … just working as normal, and then I split with my wife, got fed up from then. It was already … messy anyway, dragging out for a long time, and I've never actually been treated for depression, but I don't know if it is or not. I don't know if fed up's a, you know, a part of depression, I suppose it would be, I'm not really sure. At the moment … I'm not too bad, I've had somewhere to stay for a while, been staying at my friend's house … I'm just hoping that it will go the right way. And then once – because I will get myself sorted, it's just staying that way, that's the difficult one, that's the biggest problem I find. Just stopping … normal. I don't know if there's an answer to that or not. Bit tricky really. [Long pause]. With working, I always seem to work my way up the companies quite well, and then I mess that up sort of thing, just – I get fed up, don't turn up. I mean people have said this, it's some sort of mental health – but I've never been diagnosed, so I'm not really sure. But everything I do is always my fault. You know, I always mess ‘em up myself, and I can never understand why I do that either. Tricky really. I: Mm, sounds it, yeah. P: Yeah. I don't really know what to say now

Paul presents his experiences as “spells” of getting “fed up”; he is unclear if this is depression, but uses drugs “for that”. Things get better for a while, then worse, before he starts “get[ting] myself clean again” and back into work. He describes getting fed up again and “exactly the same thing” happening, continuing for 20 years, until getting “too old for it” appears to have prompted his approach to the service. He says he will be able to “get himself sorted” because he has in the past; the problem is “staying that way”. He identifies being fed up as starting after a messy split with his wife. He stresses that “everything I do is always my fault”.

After describing these spells, Paul comes to a halt. JLB asks some prompt questions. However, they don't seem to ‘help’ Paul to continue, and although he does say more, he keeps returning to ‘not knowing’, in what JLB perceives at the time of data collection as frustration at not being able to understand his own story. In her field notes, JLB writes that she “fears” this isn't a recovery story, as there seem to be “so many blanks and unknown things” within it.

### 3.2 Macro-level context

We identified three socio-cultural meta-narratives which could be seen as co-constructing Paul's personal account. The first speaks to the way different services in England may fail to interact. Paul is accessing substance misuse, not mental health, services; these are often separate in England. He refers throughout his narrative to uncertainty about whether he is depressed and says he has never been diagnosed or received treatment for it. Later he comments “there's got to be some mental health issue somewhere there, there's *got* to be, I mean it's insanity” [i.e. his repeated return to drug use]. However, people with MCN are often treated based on what is judged to be their ‘primary diagnosis’, with services often “designed to deal with one problem at a time and to support people with single, severe conditions” (Making Every Adult Matter Coalition, 2015: 7). Substance use can be an exclusion criteria for mental health services (House of Commons Home Affairs Committee, 2015). For Paul, these structural factors may be preventing him from accessing mental health support.

Second, another recurring thread is Paul's description of hiding difficulties from those around him. He identifies that things started to go badly for him after his marriage ended, and later comments that drinking probably masked his low mood. After a long pause he adds: “I'm always so – very good at making out there's nowt [nothing] wrong” especially to “my mam, people like that”. This extends to clinicians:

My doctors have asked me [about his mental health] – I just say I'm all right, I always have done. I don't think that’s a good thing either really, just bottling everything up, but that's what I’ve always done.

He says he doesn't like to “pass things on”, a phrase suggesting he may see his difficulties as contagious, or which may be a more palatable way of presenting reluctance to discuss difficulties with others. Here his narrative may be influenced by hegemonic meta-narratives of masculinity, characterised by emotional control and a lack of vulnerability (Emslie et al., 2006); adherence to which has been associated with depression (Parent et al., 2019) and the inhibition of help-seeking (Seidler et al., 2016). A narrative of being too old for continued “benders” may be more acceptable to him than a narrative of ‘depression’, which can be associated with powerlessness and the uncontrolled expression of emotion (Emslie et al., 2006).

A third thread relates to a moral meta-narrative; namely, that the problems of substance-users are self-inflicted and their suffering deserved. This prevailing attitude is demonstrated, for example, in Atkinson and Sumnall (2020)'s discourse analysis of substance use in the UK reality show *The Jeremy Kyle Show,* which found that users were blamed and held fully responsible for their substance use and resulting problems. Paul can be seen here as offering just such a narrative. He returns repeatedly to a stance that “everything I do is always my fault”. He marks himself as different from other people experiencing distress, saying “whenever I hear of mental health, I know that most of mine's all my own fault” and “I know mine are all self-inflicted”. He is the one who messes things up – he has brought “it” on himself. These narratives of self-responsibility, strengthened by neoliberal health policy discourses and associated austerity strategies, have been shown to “inflict, sustain and exacerbate” mental distress and suffering for people from low-income communities in a form of “narrative violence” (Thomas et al., 2020: 1125). Paul's story can be seen as evidence of Thomas and colleagues' conclusion: that such policy discourses can become naturalised and normalised by individuals themselves, and by health professionals seeking to support them; and that those with less access to material resources may be less able to resist such discourses.

This combination of meta-narratives may be seen as creating a ‘narrative deadlock’, with a materially negative effect on Paul's life. Alternatively, Paul may be shrewdly taking part in his own form of narrative resistance, deploying these meta-narratives to his own ends in exchange for something of benefit to him (perhaps the cash on offer, or the opportunity to be heard). He may be presenting exactly the kind of ‘narrative of lack’ which Lawler (2005) calls ubiquitous in the media and other discourses when describing working-class existence – a lack not simply of material resources but of “the right ways of being and doing” (Bourdieu, 2018). Paul might reasonably assume such a narrative is required of ‘people like him’ (white working class, substance-using, homeless) in the context of an interview conducted by a university researcher in a substance misuse service.

### 3.3 Micro-level context

We identified three factors related to the interactional roles of interviewer and study context which could also be seen as contributing to Paul's ‘narrative of lack’.

First, JLB's request for an *explanatory* story seems to result in Paul feeling at a loss. He presents his cycling through spells not as an active choice, but as something that puzzles him – he repeats “I don't know” and “I'm not sure” throughout. In a performance auto-ethnography of anxiety and physical activity during Covid-19 lockdowns, Carless (2022) unsettles the idea that we can always know what aids recovery. He describes his own struggle to say with confidence what works for him. He cautions: “let nobody forget that *stuff* happens that cannot be put into words” (Carless, 2022: 311, his italics). As with the ‘quantitative fallacy’, that what cannot easily be measured is not important, or does not even exist (Yankelovich, cited in Bøe et al., 2019), it may be a ‘qualitative fallacy’ to assume that what cannot be identified and put into words doesn't exist (Bøe et al., 2019). Ultimately Carless is able, as an educationally and economically resourced individual, to use narrative to “transform myself … choosing a better story for myself”. However, it may be a lot to ask of someone currently without the most basic ontological security of a home.

Second, JLB's questions and prompts are inviting a specific type of narrative. Initially, an open-ended question is used: “could you tell me about your experiences of … ?” Some guidance is then added, based on McAdams (2013)'s Life Story Interview: “can you tell me that as if it was a story?", indicating this would consist of a beginning, middle, and thoughts on present and future. However "tell me … *as if* it was a story" implies some tellings are not story-like. This introduces deductive assumptions, effectively closing down Riessman's stipulation of an ‘open-ended’ question and setting up a model template for the participant. For those whose stories do not necessarily follow such a trajectory, this model may be problematic. Paul apologises several times for not presenting his experiences in the desired way: he is “sorry it isn't really in a story” and concludes “I just wish I could put it more into a story for you”. As other studies have found, participants in narrative-based research may have negative experiences if they feel their stories do not align with what a recovery story *should* look like (see for example Nurser et al., 2018).

This ideal may also be imposed by an interviewer's normative assumptions and line of questioning. An inclusive definition of ‘recovery narratives’ was an important study principle. However, when considering JLB's prompts separately (Table 2), they show a tightly-defined idea of key components of a recovery narrative. The prompts suggest Paul's story should contain content about what helps (line 43), turning points (61) and a clear shape (107). The focus should be on how he stays positive (114) or moves on from distress (43, 86, 165, 216); on the future (134) and on advice for others (216).

**Table 2:** Paul's story (Extract 2).

Line	Prompt question
33	Where do you think it started for you?
43	What would you say helps you?
53	What does it look like when things are going well for you?
61	Does it feel like there are tipping points that tip you towards feeling “self-destructive”?
68	Why do you think that might be [bottling up difficult emotions]?
86	What sort of things have helped you be in a slightly better place?
93	What do you do [for work]?
107	If it was a story – what sort of shape would it be?
114	What I'm really struck by is that you keep going and you seem quite optimistic, and that's amazing to me […] do you know what helps you feel positive?
134	If you look to the future, what would you want?
165	Do you know what it is that changes – that gets you from one place to that next place?
173	What is next for you? What would you hope for?
216	Are there other things apart from work that you think, I would give that advice to somebody else in a similar position to me?

Two factors were driving this line of questioning. One, some un-noticed ontological assumptions derived from JLB's own experiential ‘use’ of stories. They mirror her own seeking of relief through accounts offering insights into moving on from despair (114), and maintaining a sense of hope (173). Two, an important study principle was to leave stories unedited to minimise curatorial control (Yeo et al., 2022), should the participant opt to donate their story to the NEON online intervention.These two factors are shaping JLB's attempt to elicit a sequential story with clear advice for others, in the belief this will be most beneficial. The “fear” recorded in her field notes implies concern that there is nothing in Paul's story that might be valuable for others.

This fear reflects critiques that, by creating a genre of ‘recovery narrative’ and ascribing it particular characteristics, such as inspiring hope and offering practical strategies to individuals, stories which do not fit these requirements will be excluded or not considered worthy of sharing with wider audiences (Kaiser et al., 2020). In a critical review of the mobilisation of recovery narratives in services, Woods and colleagues find that self-expression in these contexts is “highly circumscribed, goal-directed, and carefully crafted to fulfil larger imperatives” (Woods et al., 2019: 231). Here, this mobilisation is transported to the research context. A pragmatic concern with ‘what works’ may function to suppress other experiences of distress, suffering and recovery which do not conform to such templates (Pascal and Sagan, 2018). This matters, not least because, as perhaps with Paul, such pragmatism can render a person's narrative of their own experience “yet one more thing at which service users can fail” (Rose, 2014: 217). Furthermore, the stories available socially and culturally affect how others may imagine and shape their own experiences (Plummer, 2019). Analysis of JLB's implicit ontology and epistemology demonstrate how such stories may routinely become excluded from narrative interventions and research studies, if the gatekeeper (researcher, editor, curator) does not examine their assumptions about what characterises a recovery story.

A third factor co-constructing Paul's narrative is JLB's focus on a personal sense of resilience. Her expectations of recovery stories are shaping how she *hears* him, such that her questions rarely respond to what Paul is actually presenting. His story includes such potentially rich areas to explore as his marriage break-up, his relationship with his mother, his “bottling up” of feelings with family and clinicians, and his father telling him that the “only good thing” was that Paul had a trade. With a more inductive focus, interview prompts may have drawn more directly on the story he was telling. But in pursuit of an archetypal ‘recovery’ storyline, these cues were missed, and with them the opportunity to build a more contextualised picture of Paul's life.

Moreover, JLB's line of questioning means she misses something that Paul does clearly present – that it is his employment situation above anything that helps him (Table 3). JLB offers a positive reframing of what she sees as Paul's resilience ("to me that's amazing, that you don't stay there, there is something that moves you [on]..."). But this questioning appears to back him further into a corner – “I really don't know, that's the thing”. Yet when asked what is next for him, Paul is clear. He will seek out potential employers. Being good at his job helps him secure re-employment, and he mentions his employers' stance that “when you're not at work, you do what you like”. They do not judge or stigmatise, but provide supportive employment conditions where gaps are not treated as signifying a problematic employee. This is the factor Paul identifies as helping him to move on repeatedly from his phases of drug use. Thus, his story can be framed as one which indeed contains important information about recovery, but at a structural level.

**Table 3:** Paul's story (Extract 3).

I: Do you know what it is that changes – that gets you from one place to that next place? P: [Pause]. To be honest, no. Sorry! I wish I did really. It must be something! I: Sure, yeah, because to me that's amazing, that you don't stay there, there is something that moves you from that point to a point where you start doing things again. And it's fine if you don't know what it is. P: I really don't know, that's the thing. I: No, completely fine. P: I don't really know, I'm sorry. I: No worries at all. What would you say is next for you? What would you hope for? P: Well, next? I've done this a few times as well, what I keep doing is I keep wandering around the companies where I worked, one of them will see me and they'll drag me in for a coffee … and then it'll really go from there, sort of thing. I: Go from there, as in offer you some work? P: Yeah. I've done it three times, exactly the same thing. Or I'll just turn up at the pub where I know they will go for a drink every Friday. But like I say, the only good thing was, I'm good at it, and that helps, so … and also, where I work, a lot of them have, in their own frame of mind, what you do out of – when you're not at work, you do what you like. Which is, you know, which is fair enough sort of thing, as long as you come to work and do your work, that's – yeah. Yeah, so that's what I'll do next. I: Right. Yeah. P: Sorry, it isn't really in a story –

### 3.4 Cheryl: “Nothing's changed, baby”

In contrast to Paul's *narrative of lack*, Cheryl's can be read as an agentic *narrative of resistance* to the possibility of recovery. Cheryl is a white British woman in her fifties, recruited through a rights and support organisation for sex workers, where she was interviewed. Before the interview, she and her support worker discussed whether to go ahead with the interview. Cheryl was not in a good place, having visited a self-harm support organisation for the first time the day before, which involved completing a lengthy questionnaire about her experiences. She concluded she was happy to proceed as long as it wasn't “like yesterday” and opted to have her support worker present. The first extract (Table 4) is taken from the beginning of the interview.

**Table 4:** Cheryl's story (Extract 1).

I: Okay, so if you're happy to start then I'll just ask you the first question, which is that – just – erm, could you tell me about your experiences of mental health issues? And kind of how you've survived everything that has happened to you? And could you –
C: When I –
I: Sorry Cheryl, could you tell me as if it is a story? So like where you think it might have started for you? So you were just mentioning about stuff when you were little for example. And then what happened after that –
C: Well, sex … sexual and mental abuse from the age of five until 11 sexually, but mental health and the physical abuse went on till when I was 16. I was sectioned when I was 16 for three years till I was 19. I had electric shock treatment that turned me into a zombie, I was on that many tablets. And they […] [anon name], he said to me, he's only ever heard of two occasions that they've had electric shock treatment, and I'm one of them. And they only do it when you're that traumatised there's nothing left for them to do. So … I think it's shit, mate – back in them days they used to have tablets in your food and everything. And when I went on [anon ward] they just wanna fucking sedate you, if you don't do what they want, they take your own room off you and put you in a cubicle. And it's right next to the men and [inaudible].
I: Right yeah, so you didn't like that.
C: Nah. Not at all baby [pauses for a drink]. But that's about it, and I think mental health is shit to be honest. The police don't understand still. I don't think anyone understands mental health problems

Cheryl appears keen to get on with the interview, perhaps unsurprisingly considering her experiences the day before, but JLB interrupts to again request that she tell it *as if* it is a story. Cheryl opens by describing early childhood abuse, and her hospitalisation in her teens. She is angry about her hospital treatment, which she contends turned her into a sedated “zombie” and involved inappropriate accommodation for a survivor of sexual violence. She thinks mental health is still “shit to be honest”, and that the police don't understand it; no-one does. Her comment “that's about it” indicates there is nothing more to say; her story is summed up in a blunt presentation of multiple trauma and her view of poor treatment by services. Prior to recording, Cheryl describes the extent of her abuse and some of its consequences, including not being able to speak of it for many years, and continuing to experience vivid sensory flashbacks some 30 years later. During an explanation of the study's recovery context, she states she will never be able to recover; it is too late for her. In her field notes JLB describes being again unsure if this is “a story we'll consider to be a recovery narrative, by Cheryl's terms”.

### 3.5 Macro-level context

Two contrasting meta-narratives of mental health can be seen in Cheryl's story, as illustrated here:

C: I start counselling this month on [day], about it

I: Have you had any counselling before?

C: Yeah, but I didn’t open up

I: Do you know why that was?

C: Too painful, babe

I: Yeah. Yeah, sure

C: But I’ll do it, cos that’s the only way I’m gonna move on, innit? I can’t keep living like that, it’s – but I know I’ll always have depression, I know I’ll always cut myself, because it’s release. I don’t do it for sympathy, I don’t do it when anyone else is in the house, but as soon as I see that blood it’s like the whole world’s been took off my shoulders.

Arguably, Cheryl employs the vocabulary of personal resilience common to recovery accounts here, seeing herself as needing to “open up” and “move on”. She will participate in counselling despite anticipating a painful process. On the other hand, a narrative of resistance can also be seen – she will “always” have depression and thus cut herself. She says it is not sympathy she wants, but release.

Before the interview, Cheryl actively refutes her story as one of recovery. She has experienced formal, coercive mental health treatment, which she reports has not helped. She may want, at least in part, to access counselling, but Cheryl does not see herself as mentally ill or mad, as indicated by her later distinguishing of herself from other inpatients: “the second time I was sectioned, if I didn't do what they said, they took my own room off me and put me in a cubicle with a lunatic”. This is not a story of seeking a diagnosis. Her account can be seen as one of trauma and abuse; her self-harm a reasonable response (rather than an absence of recovery), which requires no further explanation.

Nor is Cheryl's story a moral tale about escaping sex work. There is no mention of Cheryl's current life circumstances, perhaps indicating her active selection of topics she considers relevant for the interview. Her focus can be seen as remaining with structural causes of her distress as she sees it: childhood abuse, coercive mental health services, treatment by uncomprehending police and ongoing intimate partner violence. Her agentic responses to ongoing trauma include self-harm and suicidal thoughts, and a willingness to try counselling and access support from the rights organisation. This shaping of her experience mirrors a meta-narrative of a trauma-informed approach to mental health, wherein mental distress is constructed not as a disorder located within a person, but a rational response to, and communication about, structural injustice (Sweeney et al., 2016). Cheryl's narrative is co-constructed in the context of support from a rights-based organisation, working to challenge inequality as well as provide immediate support. Ironically, her resistance to a recovery discourse arguably returns the genre of ‘recovery narratives’ to a focus on one of the original emancipatory concerns of survivor groups who told them: namely, attention to human rights and the structural causes of distress.

### 3.6 Micro-level context

Three immediate-context factors can also be seen as co-constructing Cheryl's narrative. First, JLB's discomfort with a recovery framing, in this context. Following Cheryl's pre-interview description of continued trauma, JLB chooses to drop the word ‘recovery’ from her opening question, as it seemed inappropriate. Her discomfort can be seen in the hesitation and slight stumbling before asking if Cheryl can talk about her “experiences of mental health issues”, and the subsequent phrase “how you've survived everything that's happened to you”. In response, Cheryl repeats the details of her abuse without hesitation. The ease and free sharing of intimate details with a stranger may suggest she is accustomed to sharing her story with others, and is adept at doing so. She may be used to giving of her intimate self to professionals; as a sex worker she may not be afforded the luxury of privacy in many parts of her life.

Her readiness to provide such information may also indicate that she does not want prolong the experience. In contrast with Paul, she is agentic within the interview. She has already been asked two questions and does not need the framing of “tell me as if it is a story”. She appears confident in her responses and in selecting what she tells, as well as dictating the terms and length of the interview. She focuses on what she may assume is required – the traumatic details. Given her weariness from the day before, she may also want a no-nonsense exchange of her story as quickly as possible for the promised cash.

Second, JLB's pursuit of a personally transformative turning point elicits an embodied refutation from Cheryl. JLB attempts to prompt a linear account of Cheryl's earlier life:

I: Do you remember what happened next in your life after being in the hostel?

C: Nothing's changed, baby. I still feel the same, I still wanna kill myself every day. I still self-harm, I done that a couple of weeks ago [shows scars]. It's always deep, when I do that down my arms, it sends electric shocks. Because I’ve damaged the nerves in my arms, cos that's how deep I cut.

I: Deep, yeah right –

C: Get me, nothing's changed mate … not nothing, no one understands …

As with Paul, JLB is pursuing a traditional story arc with a turning point, but this does not resonate with Cheryl. Nothing has changed, she says; she still wants to kill herself every day. She shows JLB the scars on her arms and emphasises the nerve damage caused due to the depth of her cutting. It is as if, having told what she thinks she is expected to share, she does not elaborate but instead embodies her trauma with a physical demonstration of the depth of her distress. The academic literature on self-harm, despite widely seeing it as a response to trauma, generally portrays it as a failure to develop healthy coping mechanisms (Nock, 2009; Favazza, 2011). However Cheryl's openness can be read in another, more agentic way. Gurung (2018: 35) suggests that “those who engage in self-harm practices are performing embodied, socially situated acts of healing, survival, and self-creation in a physical attempt to retell complex, fragmented stories of abuse, existential angst, trauma, and loss of self”. Cheryl's physical embodiment of her trauma may illustrate this, in what can be seen as an act of resistance to cognitively ‘sanitising’ her story (Costa et al., 2012), with linear plots, defined turning points and neat, happy endings.

Third, we see JLB's positive reframing which clashes with Cheryl's continued sense of violation. The tone of the interview changes after JLB attempts to reframe Cheryl's suicide attempt:

C: Maybe that was God's will, you get me? Maybe not to jump.

I: Yeah, maybe – yeah.

C: And I don’t – I don’t understand anything anymore.

I: Mmmm. But you’re still here.

C: Life’s just a bitch, isn’t? I’ve had nothing but violent relationships, apart from [partner] and then he died when he was 35 through the alcohol, that’ll be twelve years at end of August. He’s the only guy what’s tret me right, in my – since I was a kid. [Inaudible] I’ve always fallen for guys that abused me, hit me. Always. And then, I have to move on from that.

[Long pause. Cheryl cries, interviewer offers tissues]

I: Shall we stop?

C: Move on to the next question please.

JLB is here offering affirmation of Cheryl's ability to survive ("but you're still here"). However this does not land with Cheryl, as she reflects on the violence in her adult relationships. Her voice and body language soften as she talks about “the only guy what's tret [treated] me right”, and cries. JLB moves on as requested, asking whether others' stories have helped her. Cheryl says no, they upset her, and repeats that she hasn't got long. JLB asks whether other people's stories have ever been unhelpful. Cheryl replies “baby, no-one's been through my life”. She shows other scars on her arms, not of self-harm, but from her childhood abuse, describing who and what made them. In response to JLB's continued questions, she returns to the physical evidence of her abuse, co-situated with her own sites of self-harming; an embodiment of her trauma and survival when perhaps language has proved inadequate.

The interview ends when Cheryl says she continues to feel violated “by everything”. Her shift from instructional and matter-of-fact to tearful raises a number of questions on the nature of the exchange. Why did Cheryl consent to the interview, given her experiences the day before, and that she does not consider hers a story of recovery? As is considered good ethical practice, participants' labour was recompensed by offering vouchers. Although the alternative seems worse and has been critiqued as exploitative of lived experience narratives (Yeo et al., 2022), payment runs the risk here of rendering the exchange straightforwardly transactional; cash for trauma. By offering a financial incentive, is Cheryl giving what she thinks is required as quickly as possible, despite the cost to herself, thereby mirroring her sex-working relationships? Or is this a reductionist analysis? Whatever her reasons, what was seen at the time of data collection as a sparse narrative becomes rich when viewed as an embodiment of the fractured and stark nature of her trauma, and a refusal to sugar-coat ongoing experiences of distress in the face of continued structurally unjust conditions.

## 4 Concluding discussion

Our findings can be summarised at two levels. First, we explored the kinds of recovery stories people with MCN may tell. Paul can be seen as constructing a *narrative of personal lack*, blaming himself for his situation and apologising for his inability to give a storied account. In contrast, Cheryl can be seen as constructing a *narrative of resistance*, rejecting a label of mental illness and any possibility of recovery. Paul and Cheryl give very different accounts, but ‘recovery’ appears to have little meaning for either in their current situations. Neither presents the kind of ‘paradigmatic’ narrative of recovery involving a ‘transformed Self’, with a starting point, ongoing process and ultimately transformed life situation (Hydén, 1995). Mental health inequality is a complex phenomenon with interacting micro and macro level components (Karadzhov, 2021). Yet recovery research has been criticised for its over-focus on the agentic level of identity transformation or resilience, at the expense of structural factors (Padgett et al., 2016; Harper and Speed, 2012). Critics of the ‘recovery narrative’ point to the same issues with their elicitation and reception (Voronka, 2019; Woods et al., 2019). Our findings provide empirical evidence of this over-emphasis in action, demonstrating how the elicitation of recovery narratives can mirror this focus on individual resilience and attempts to ‘overcome’. This may function to occlude the structural causes of mental distress, as well as the heterogeneous ways in which people endure or carry on without expectations or experiences of transformation.

Attention to structural factors within narratives (including those within the ‘hearer’) is vital to ensure that recovery research does not continue to maintain a ‘blind spot’ in this area (Topor et al., 2021). Offering decontextualised, reductionist forms of recovery narrative which pay insufficient attention to the economic, institutional and political injustices that people experiencing mental distress may systematically endure does little to address the needs of the most vulnerable (Karadzhov, 2021; Morrow and Malcoe, 2017).

Second, and ironically, our findings function as a critique of the very process of eliciting and using recovery narratives as a source of knowledge. Like others, we are “leveraging the methodology I am questioning”, as Burkette (2022) describes in a performative reinterpretation of the research interview. Narrative researchers can think ourselves the ‘good guys’, dealing with meaning, purpose and attempts to counter dominant narratives (Costa et al., 2012). However, this is not enough to ensure genuinely emancipatory research. We may be the ones who by our own self-reflexivity have figured out how to be *really* effective at stealing stories (Church, 2013). Nor is being a researcher with lived experience sufficient to ensure an epistemically just approach. As Russo (2016) states, other structural inequalities “affect the aspiration to such a ‘we’". She cites Collins who, in advocating for intersectional scholarship, reminds us that knowledge cannot be separated from the power relations which shape it (Collins, 2012).

Research is about power in the mundane practices too. The ‘big stories’ of the research proposal and ethics protocol provide the context for the more intimate encounter of the interview, and shape how these will be understood (Church, 2013). As Bourdieu has it, in order to objectify the social conditions of the production of knowledge, researchers must “turn the instruments of knowledge that they produce against themselves, and especially against the social universes in which they produce them” (Bourdieu, 2000: 121). By adopting a critical and reflexive standpoint, it was possible to examine our own epistemological assumptions at all levels of the study, revealing the ways in which a pragmatist approach to research (Rorty, 1999) can result in unacknowledged assumptions being embedded into the design. We found that our research methods, despite social justice-oriented intentions, were inadvertently reproducing a neoliberalist agenda (Pascal and Sagan, 2018). Recovery is epistemologically individualist. It appears to be neutral and accessible to all – but it is not and will not be unless the structural determinants of health are taken seriously.

What then, for narrative-based research in the field of mental distress? Russo suggests giving up analytical aspirations to interpretive dominance. She echoes Frank (2010) in seeing interpretation instead as an ongoing dialogue with the story which recognises the ‘unfinalisability’ of other people. Church offers six thoughts on what politically alert researchers can do, including complicating what we are listening for – “less for stories of healing and recovery and more for stories of resistance and opposition" (Church, 2013: 29). Pascal and Sagan (2018) call for making the ‘outlier’ narratives our core business. In choosing to foreground, not omit, stories which do not fit the neat template of ‘recovery narrative’, we hope to have contributed to the continued hearing of voices which might be silenced in this field.

We also hope to have further complicated the concept of ‘recovery’. Research on ‘recovery narratives’ which is simplified and stripped of context risks reinforcing neoliberal ideas of individual responsibility for their own wellbeing for some of the most structurally disadvantaged people in society, while leaving living conditions, and ongoing situations of social injustice, unchallenged and unchanged. A critical, reflexive approach, together with transparent researcher positionality, is imperative to avoid the epistemic injustice of decontextualised forms of recovery narrative.

## Credit author statement

**Joy Llewellyn-Beardsley**: Conceptualisation; Investigation; Methodology; Data curation; Formal analysis; Writing – original draft; Writing – review & editing. **Stefan Rennick-Egglestone**: Data curation; Writing – review & editing; Supervision; **Felicity Callard**: Formal analysis; Writing – review & editing. **Kristian Pollack**: Formal analysis; Writing – review & editing. **Mike Slad**e: Funding acquisition; Writing – review & editing. **Alison Edgley**: Formal analysis; Writing – original draft; Writing – review & editing; Supervision.

## Data availability

An anonymous version of the transcripts underpinning this publication is available on reasonable request from the study sponsor, Nottinghamshire Healthcare NHS Foundation Trust, but only for those individual participants who have provided written consent for use in secondary analysis outside of the NEON study. Field notes will not be released. Obtaining access requires an institutional signature on a generic end user license and a set of special licensing conditions. Contact the study sponsor through research@nottshc.nhs.uk.

## Declaration of competing interest

The authors declare that they have no known competing financial interests or personal relationships that could have appeared to influence the work reported in this paper.
