# Supplementary material for: “Nothing's changed, baby”: How the mental health narratives of people with multiple and complex needs disrupt the recovery framework
Source: SSM Ment Health. 2023 Dec;3:100221. doi: 10.1016/j.ssmmh.2023.100221 (PMC10316065; doi:10.1016/j.ssmmh.2023.100221)
Supplement: Multimedia component 1 [file mmc1.docx]

# Supplementary Material 1

**Participant Information Sheet - Activity 1.**

<Anonymised study information>.

# Supplementary Material 2

# Topic guide

PART A (UP TO 45 MINS)

1. Can you tell me in your own words about your mental health and recovery experiences please? For this first part of the interview, I don’t have any set questions to ask you – could you tell me about your experience as if it were a story with a beginning, a middle and how things might look in the future? There’s no right or wrong way to tell your story – just tell me in any way that feels most comfortable.

*Prompts: (only if narrator comes to a stand-still): Can you tell me more about that? What was the experience like for you?*

*Is there anything else you’d like to tell me about your recovery story?*

PART B (UP TO 45 MINS)

2. How was that experience for you, telling me something about your story today?

*Prompts: is it something you’re really familiar with doing, or not?*

3. How do you vary how you talk about your experience, depending on the context? E.g who you’re talking to, where you are, how you’re feeling that day?

*Prompts: Have you ever felt that there are parts of your story that you’re unable to share in a certain context?*

4. As you know, we’re interested in how stories might affect people who hear them, and we’re aware that sometimes it might NOT be helpful to hear a story of someone else’s experience. Can you think of examples of times when people sharing their experiences have been unhelpful to you?

*Prompts: Can you tell me more about this? What was unhelpful?*

*Prompts: What were your personal circumstances at the time?*

5. And can you think of examples of stories that have been helpful to you?

*Prompts: Can you tell me more about this? What was helpful?*

*What was it about the person or story that had the impact? / What were your personal circumstances at the time?*

Has this changed the way in which you give your own accounts in an attempt to help others?

*Prompts: Can you tell me more about this? What was helpful?*

# Supplementary Material 3

# Performative narrative analysis template document

*Based on Bamberg’s (2020) integrative approach and Bengtsson & Andersen’s (2020) performative approach. Produced by [Anon] and [Anon] in 2022. Permission to re-use this template is given, under the terms of the Creative Commons CC-BY license.*

*Cite as: [Anon}*

**Stage 1 analysis (Bamberg’s identity navigation questions)**

1.1 How does the participant present themselves in the interview?

1.1.1 SAMENESS/DIFFERENCE: How does the participant present themselves as different, similar or the same with respect to others?

1.1.2 AGENCY/PASSIVITY: How does the participant navigate between agency (capable of producing and changing things in their world) and/or passivity (recipients of biological/natural or social forces) in how they presents their story?

1.1.3 CONTINUITY/CHANGE: How does the participant navigate aspects of continuity and/or change in their story?

**Stage 2 analysis (Bamberg/Bengtsson & Andersen questions combined)**

**2.1 Immediate interpersonal context**

2.1.1 How does the participant position themselves in relation to the interviewer/ wider audience?

2.1.2 How does the interviewer actively participate in both production and ongoing interpretation of narrative?

**2.2 Socio-cultural context**

2.2.1 How does the participant position themselves in relation to dominant discourses?

2.2.2 How is the immediate context linked to broader historical/socio-cultural contexts?

2.2.3 How do these have an impact on expectations of what must be explained and what is self-explanatory?

2.2.4 What does the participant *not* explain?
